# *Toxoplasma gondii* Chromodomain Protein 1 Binds to Heterochromatin and Colocalises with Centromeres and Telomeres at the Nuclear Periphery

**DOI:** 10.1371/journal.pone.0032671

**Published:** 2012-03-09

**Authors:** Mathieu Gissot, Robert Walker, Stephane Delhaye, Ludovic Huot, David Hot, Stanislas Tomavo

**Affiliations:** Center for Infection and Immunity of Lillle, CNRS UMR 8204, INSERM U 1019, Université Lille Nord de France, Institut Pasteur de Lille, Lille, France; University of Melbourne, Australia

## Abstract

**Background:**

Apicomplexan parasites are responsible for some of the most deadly parasitic diseases afflicting humans, including malaria and toxoplasmosis. These obligate intracellular parasites exhibit a complex life cycle and a coordinated cell cycle-dependant expression program. Their cell division is a coordinated multistep process. How this complex mechanism is organised remains poorly understood.

**Methods and Findings:**

In this study, we provide evidence for a link between heterochromatin, cell division and the compartmentalisation of the nucleus in *Toxoplasma gondii*. We characterised a *T. gondii* chromodomain containing protein (named TgChromo1) that specifically binds to heterochromatin. Using ChIP-on-chip on a genome-wide scale, we report TgChromo1 enrichment at the peri-centromeric chromatin. In addition, we demonstrate that TgChromo1 is cell-cycle regulated and co-localised with markers of the centrocone. Through the loci-specific FISH technique for *T. gondii*, we confirmed that TgChromo1 occupies the same nuclear localisation as the peri-centromeric sequences.

**Conclusion:**

We propose that TgChromo1 may play a role in the sequestration of chromosomes at the nuclear periphery and in the process of *T. gondii* cell division.

## Introduction

Apicomplexan parasites are responsible for some deadly parasitic diseases affecting humans and live stock. They comprise a wide range of unicellular eukaryotes among which *Plasmodium falciparum* and *Toxoplasma gondii* are the most serious threat to human health. *T. gondii* is responsible for encephalitis in immunocompromised individuals and birth defects in the offspring of infected mothers. The genetic tractability of *T. gondii* makes it a useful model for the study of apicomplexan parasites [1]. The life cycle of *T. gondii* is complex with multiple differentiation steps that are critical to the survival of the parasite in human and feline hosts [2].

In six hours, *T. gondii* tachyzoites of the most virulent Type I strain perform mitosis, assembly of the cytoskeleton and membranes that form the pellicle, loading of the growing bud with organelles, and finally, emergence of new, fully formed, invasive daughters from the mother cell [3]. *Toxoplasma* tachyzoites replicate by closed mitosis named endodyogeny where the nuclear membrane remains intact throughout the cell cycle [3]. The parasites display an unusual three replication phases G1, S and M while the G2 is apparently absent [4]. Whereas expression profiles change dramatically during cell cycle [5] and differentiation [6], the molecular mechanisms controlling gene expression are still poorly understood in apicomplexan parasites.

The eukaryotic genome is structurally separated into ‘low transcriptional activity’ heterochromatic regions and more permissive euchromatin regions [7]. The identification of a wide array of *T. gondii* enzymes modifying or remodelling chromatin [8] and the discovery of a specific set of chromatin marks [9] at the active promoters suggest an important role for chromatin structure in gene regulation. Nevertheless, much less is known about the epigenetic contours of heterochromatin in *T. gondii*. In *Schizosaccharomyces pombe* and other eukaryotes, heterochromatin is defined by the presence at specific genomic loci of H3K9me modified histones and a global hypo-acetylation of histones [7]. H3K9me2, H3K9me3 and the proteins encompassing a chromodomain (CHRomatin Organisation MOdifier) that are able to bind to these modified histones are essential for genomic integrity and are localised at centromeres, telomeres and repetitive sequences [7]. Different chromodomain proteins exhibit different binding affinities to specific modified histones [10]. Proteins preferentially binding to H3K9me3 peptides are of the HP1-like (Heterochromatin protein 1) family and proteins that have a higher affinity for H3K27me3 peptides [10] are of the Polycomb family. HP1-like chromodomain proteins act as links between the H3K9me3 labelled chromatin and effectors participating in chromosomal processes such as transcriptional silencing, cell division and nuclear organisation [7], [11]. Recently, the H3K9me2 and H3K9me3 marks were identified at the peri-centromeric regions [12], defining the first heterochromatic regions in the *T. gondii* genome. Surprisingly, the H3K9me histones were not enriched at promoters of developmentally regulated genes and regions of the genomes that are constitutively silenced in the intermediate host [12]. In *P. falciparum*, H3K9me3 and its binding protein, PfHP1, were mainly localised at the subtelomeric regions but not at the centromeres [13]. In order to understand the role of heterochromatin in *T. gondii* gene regulation and genomic integrity, we characterised a chromodomain containing protein (TgChromo1). In this study, we show that TgChromo1 specifically binds to hetechromomatin in *T. gondii* and that it may have an important role in centromere biology. In addition, we report that TgChromo1 colocalises with centromeric and telomeric sequences at the nuclear periphery, suggesting a role in the nuclear organisation during the cell cycle of *T. gondii*.

## Results and Discussion

### Identification and functional characterisation of TgChromo1

**Figure 1:**
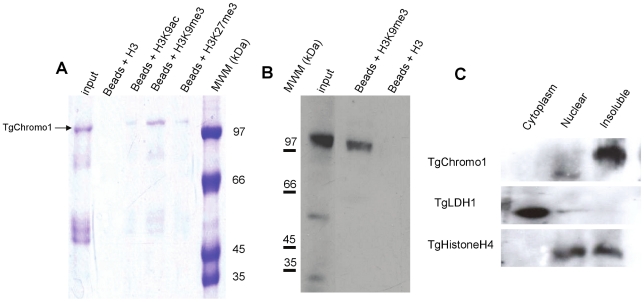
TgChromo1 is a chromodomain containing protein. **A:** TgChromo1 specifically binds to H3K9me3. TgChromo1 protein was bound to H3K9me3 peptides fixed on Agarose-beads. The binding was then competed by excess amount of different H3 peptides. A fraction of the input protein sample was loaded along with elution with an unmodified H3 (Beads+H3), H3K9ac (Beads+H3K9ac), H3K9me3 (Beads+H3K9me3) or H3K27me3 (Beads+H3K27me3) peptides. Molecular weight markers (kDa) are indicated on the right. **B:** Native TgChromo1-HA protein binds to H3K9me3 peptides. Extracts from the TgChromo1-HA RH ΔKu80 strain were purified using either H3K9me3 peptide bound beads or unmodified H3 bound beads. Eluates were subjected to Western-blot using anti-HA antibody to reveal the presence of TgChromo1-HA. Molecular weight markers (kDa) are indicated on the left side of the panel. **C:** The majority of TgChromo1 is co-purified with insoluble material. Cytoplasmic, nuclear and insoluble extracts from the TgChromo1-HA RHΔKu80 strain were subjected to a Western-blot using the anti-HA specific antibody. A control, a cytoplasmic resident (TgLDH1) and a nuclear resident (TgHistone4) were also identified in the same extracts.

HP1-like proteins are characterised by the presence of an N-terminal chromodomain that specifically binds to H3K9me3 [10]. There are three predicted genes encoding such a domain in the *T. gondii* genome (TGME49_058240, TGME49_068280 and TGME49_069760). We decided to characterise the TGME49_068280 gene, which encodes a protein containing a putative chromodomain that had the highest similarity to the HP1 ortholog in *P. falciparum* [14] and other eukaryotes (Figure S1). We accordingly named this protein TgChromo1. To confirm that TgChromo1 has the functional properties of HP1-like chromodomain proteins, we expressed a recombinant protein containing the chromodomain of TgChromo1 (amino acid from 554 to 1084 of TGME49_068280 in *E. coli*). The purified TgChromo1 protein was then used for pull-down assays where we showed that it is able to bind to beads coated with a peptide representing H3K9me3 (Figure 1A). To further characterise the specificity of these interactions, we competed the binding with excess of unmodified H3, H3K9ac, H3K9me3 and H3K27me3 peptides and demonstrated that TgChromo1 has the highest affinity for H3K9me3 peptides (Figure 1A). In order to study the endogenous TgChromo1 protein, we performed a knock-in in the RH ΔKu80 genetic background [15] replacing the endogenous copy of the gene with an HA tagged version (Figure S2). To verify that the endogenous TgChromo1 protein was also able to bind to H3K9me3, we purified proteins using either H3K9me3 peptide beads or un-modified H3 peptide beads. Accordingly, we pulled-down the endogenous TgChromo1 protein as confirmed by Western blot. In this case, the anti-HA antibody recognized the endogenous TgChromo1 after purification on the H3K9me3 beads, whereas no protein was pulled down by the H3 peptides (Figure 1B), thereby confirming that the endogenous protein has all the functional characteristics of a genuine HP1-like chromodomain protein. Similar to other HP1-like proteins [16], we mainly identified TgChromo1 in the insoluble fraction of parasite nuclear extracts (Figure 1C), suggesting that this protein belongs to the highly-dense, heterochromatic structure or is associated with the nuclear membrane. As a control for the cellular fractionation, we identified in the same fractions a cytoplasmic resident (TgLDH1) and a nuclear resident (TgHistoneH4) also found associated with chromatin in the insoluble extract (Figure 1C). Taken together, the data presented here show that TgChromo1 has all the biochemical features required for a chromodomain protein belonging to the HP1-like chromodomain family.

### TgChromo1 localises to discrete nuclear foci and its expression is cell cycle-regulated

**Figure 2:**
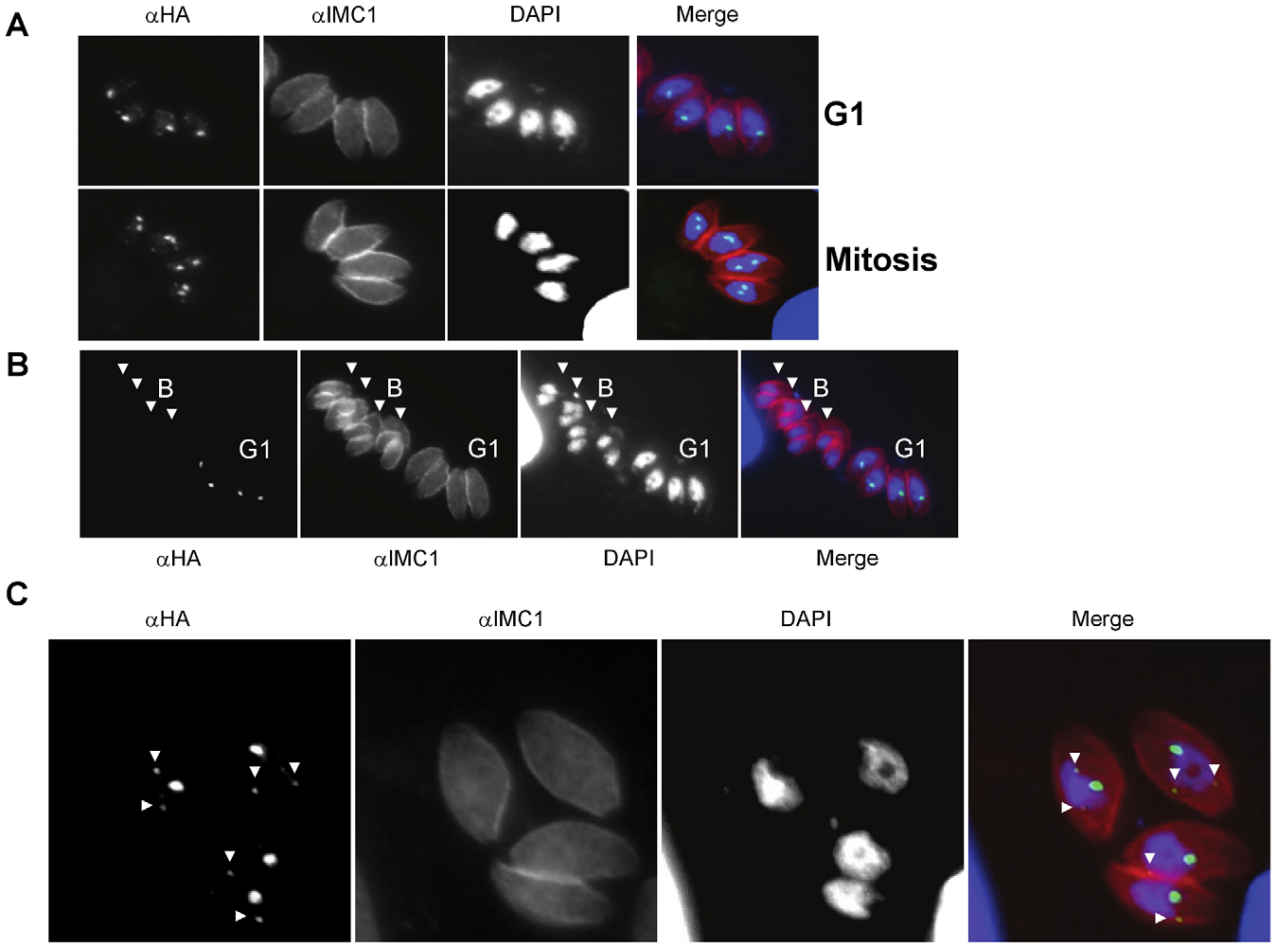
TgChromo1 expression is cell cycle regulated. **A:** IFA of TgChromo1-HA (green) and IMC1 (red), a marker of the inner membrane complex, throughout the cell cycle. Parasites representative of interphase (G1) and mitosis are presented. Parasite nuclei are labelled with DAPI. **B:** Comparison of the intensity of the signal produced by IFA of TgChromo1-HA in interphase (G1) and during budding (Budding, B). IMC1, a marker of the inner membrane complex, is used to identify emerging daughter cells during the budding. Parasites during budding (B) are arrowed. Parasite nuclei are labelled with DAPI. **C:** TgChromo1 is concentrated in foci of different intensity. IFA was performed using an anti-HA antibody and the IMC1 antibody. Parasites nuclei are labelled with DAPI. Foci of lesser intensity are arrowed.

Using the TgChromo1-HA strain and a cell cycle marker (TgIMC1), we showed that TgChromo-1 maintains a nuclear localisation throughout the tachyzoite cell cycle (Figure 2A). Interestingly, TgChromo1 is concentrated in one or two spots in the nucleus depending on the point of the cell cycle (Figure 2A). The number of TgChromo1 foci strictly follows the increase of DNA content during replication, one in G1 and two during mitosis (Figure 2A). However, TgChromo1-HA is undetectable at the end of budding or at the onset of G1 (Figure 2B), as well as in extracellular parasites (Figure S3) that are, in majority, at the G0 phase [17]. The polyclonal antibody raised against the TgChromo1 recombinant protein confirmed these features (Figure S4). This profile is in concordance with the TgChromo mRNA steady state level measured by microarray [5]. Interestingly, the parasites are undergoing a main switch from mother to daughter at the onset of G1, a time where they are more able to egress and invade [17]. TgChromo1 might participate in this switch during the parasite cell cycle by virtue of its own regulated expression and serve as one of the mitotic check-points for exiting budding and entering G1.

We also discovered that in G1, and also at the beginning of S phase, TgChromo1 concentrates as one or two additional spots of a much weaker intensity (Figure 2C, arrowed). Opposite to the main foci of stronger intensity, TgChromo1 is also present as smaller and distinct foci that have a perinuclear localisation (Figure 2C). This observation is confirmed by the three-dimensional reconstruction (Movie S1). These additional spots are not visible in Figure 2A because they are of much weaker intensity than the main stronger foci.

### TgChromo1 is maintained near the centrosome and the centrocone throughout the cell cycle

**Figure 3:**
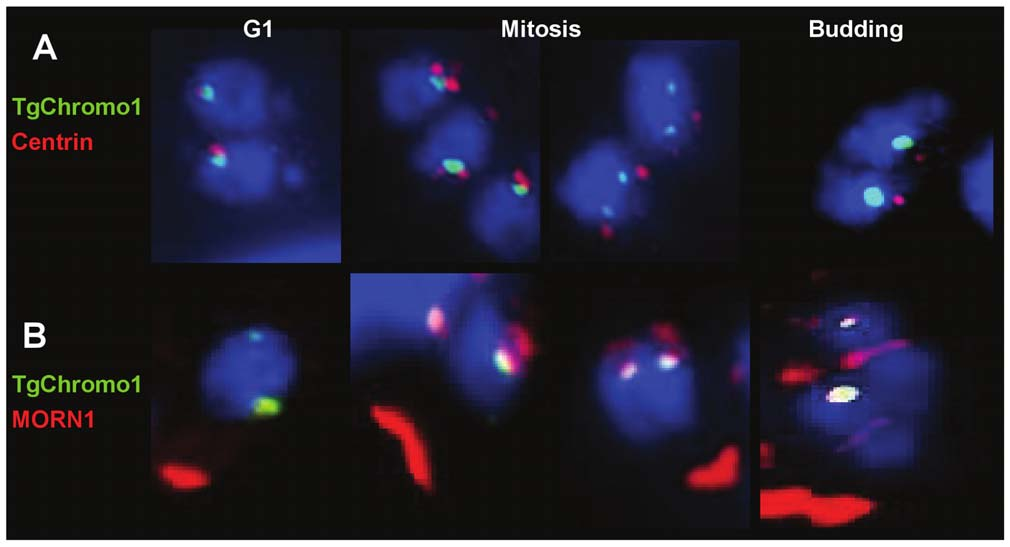
TgChromo1 is maintained near the centrosome and the centrocone throughout the cell cycle. **A:** IFA of TgChromo1-HA (green) and centrin1 (red), a marker of the centrosome. Parasite nuclei are labelled with DAPI (blue) at the interphase (G1), mitosis and the beginning of budding. **B:** IFA of TgChromo1-HA (green) and MORN1 (red), a marker of the centrocone. Parasite nuclei are labelled with DAPI (blue) at the interphase (G1), mitosis and the beginning of budding.

To better characterise the nature of the structures containing TgChromo1 in the nucleus, we used antibodies specific to two markers of the cell division, Centrin1 and MORN1, together with TgChromo1 by IFA. *Toxoplasma* spindle poles are embedded in the nuclear envelope in a distinct, electron dense membrane invagination termed the centrocone [4]. MORN1 is a molecular marker of the centrocone, while Centrin1 is a component of the centrosome [4]. At G1 phase, TgChromo1 is in proximity of the centrosome and follows its division and movement to the apical end of the nucleus during mitosis (Figure 3A). At all times of the cell cycle, TgChromo1 colocalises with the nucleoplasm side of the centrocone as represented by MORN1 staining (Figure 3B). These data, together with the localisation of H3K9me3 at the peri-centromeric chromatin [12], suggest that TgChromo1 may participate in the sequestration and movement of centromeres throughout the parasite cell cycle. Moreover, TgChromo1 dynamics in the nucleus follows the same features of a marker of the centromere, CenH3 [12]. Interestingly, after the division of the centrosome (corresponding to the metaphase of mitosis), the signal given by TgChromo1 is elongated when the newly divided centrosomes are separated (Figure 3A, Mitosis). It is worth to notice that this localisation is different than the centromeric Histone variant CenH3 at the same stage [12].

### TgChromo1 is localised to the peri-centromeric sequences

**Figure 4:**
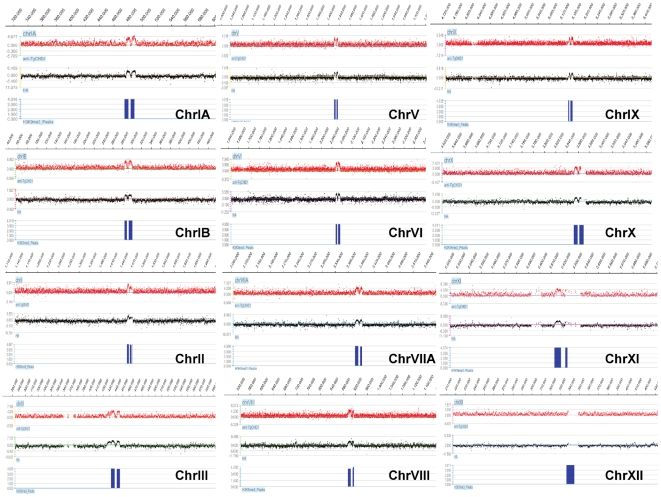
TgChromo1 binds to peri-centromeric heterochromatin. ChIP on chip was performed with the TgChromo1 antibody (anti-CHD1, red) or the anti-HA antibody (HA, black) and hybridized on a genome-wide tiling microarray. The regions of enrichment for H3K9me3 [12] are represented in blue. A snapshot of the 12 chromosomes where a centromere was identified [12] is presented. ChIP on chip signals are represented as a log2 ratio of the signal given by the immunoprecipitated DNA over the input and plotted according to the genomic position of the oligonucleotide.

To identify the genomic regions targeted by TgChromo1, we created a tiling microarray representing all the genomic sequences of the *T. gondii* reference strain (ME49). Chromatin immunoprecipitation was performed using either a polyclonal antibody raised against the TgChromo1 recombinant protein that recognize a single band in Western blots (Figure S5) (Figure 4, in red) or a monoclonal HA antibody (Figure 4, in black). The two profiles were compared to the peaks of H3K9me3 (Figure 4, in blue) previously identified for 12 of the 14 chromosomes using a similar technique [12] and available on ToxoDB. As shown in figure 4, TgChromo1 chromatin is remarkably enriched at the peri-centromeric sequences where H3K9me3 was previously identified. These data show that TgChromo1 binds to the peri-centromeric sequences and is a member of the complex of proteins forming heterochromatin in *T. gondii*.

In contrast to eukaryotic peri-centromeric sequences, which are characterised by repeats [7], more than half of the peri-centromeric heterochromatic sequences identified in this study lacked repeat sequences. Chromosome II, V, IX, XI and XII contain repetitive sequences of limited size (around a 1000 bp repeated 2–5 times), although they have no similarity to each other. Together with the lack of similarity between peri-centromeric sequences among *Toxoplasma* chromosomes, our data suggest that peri-centromeric regions are probably defined by their epigenetic context. In contrast, sequence identity is crucial to defining *Plasmodium* centromeres [12] in good concordance with the absence of H3K9me3 and PfHP1 at the peri-centromeric sequences [14], underlining clear variations in centromere biology among different Apicomplexa.

We assessed the enrichment of TgChromo1 at known silenced genes and confirmed that TgChromo1 is not enriched at their promoter sequences, in concordance with the lack of H3K9me3 enrichment at these regions [12]. Quantitative PCR was performed on peri-centromeric and centromeric chromatin of three chromosomes (Ia, V and VIII), as well as at the promoters of expressed (*eno2*) and silenced (*eno1*) genes and confirmed the presence of TgChromo1 at peri-centromeric heterochromatic regions but not at centromeric regions (Figure S6). Therefore, TgChromo1 may not be involved in the silencing of developmentally regulated genes. This is in concordance with the enrichment of H3K9me3 and H3K9me2 mainly at peri-centromeric chromatin [12].

### TgChromo1 and peri-centromeric sequences are co-localised in the nucleus

**Figure 5:**
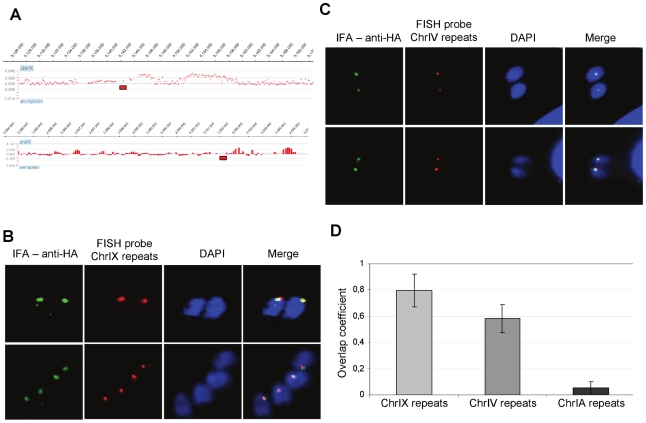
Colocalisation of TgChromo1 and peri-centromeric sequences and identification of a missing centromere on chromosome IV. **A:** Genomic localisation of the FISH probes. Fish probes (red rectangles) approximate locations are plotted against the signal of the TgChromo1-HA ChIP on chip. **B:** FISH/IFA of TgChromo1-HA (green) and chromosome IX peri-centromeric repeats (red). Parasite nuclei are labelled with DAPI (blue). **C:** FISH/IFA of TgChromo1-HA (green) and chromosome IV putative peri-centromeric repeats (red). Parasites nuclei are labelled with DAPI (blue). **D:** Quantification of the overlap coefficient between the signals of FISH and IFA. The coefficient is expressed as a ratio of the number of pixel overlapping in IFA over FISH in a given area.

In order to verify that the peri-centromeric sequences were associated with the TgChromo1 nuclear foci identified by IFA, we developed Fluorescence in Situ Hybridization (FISH) method in *Toxoplasma*. A probe representing a repeated sequence in the peri-centromeric heterochromatin of chromosome IX (Figure 5A) was labelled and hybridized with fixed *T. gondii* parasites, followed by IFA using the anti-HA antibody (Figure 5B). As shown by the quantification of overlapping signals for TgChromo1 and the chromosome IX peri-centromeric repeats (Figure 5D), TgChromo1 localises at the same region in the nucleus as peri-centromeric sequences. These results, obtained by loci-specific FISH, are in a good agreement with the ChIP-on-chip data. Furthermore, they provide, for the first time, a direct confirmation that the peri-centromeric sequences are sequestered throughout the cell cycle to a specific nuclear region and illustrate a functional organisation of chromosomal domains in the *Toxoplasma* nucleus.

Centromeres of only 12 out of 14 *Toxoplasma* chromosomes were identified in a previous study due to the limitation of the ChIP-on-chip technology, which is based on the genome assembly [12]. We took advantage of our established FISH technique to test candidates for peri-centromeric sequences after scanning the two remaining chromosomes for regions of weak TgChromo1 enrichment. One such region was identified at the very end of chromosome IV (Figure 5A) and was tested by FISH and IFA. As shown in Figure 5C, there is a significant overlap of the signals from IFA and FISH, thus demonstrating that this region of the chromosome is likely to encompass the centromeric sequences for chromosome IV. We measured the overlap coefficient for the IFA and FISH signals and showed that both tested regions (chromosome IX and IV) were significantly overlapping in contrast to a probe representing the telomeric repeats of chromosome IA (Figure 5D), although the putative centromeric sequences of chromosome IV show a lesser degree of overlapping signal with TgChromo1. Therefore, we conclude that combining the high-throughput ChIP-on-chip technology with FISH technique in *T. gondii* was useful for the proper localisation of the chromosome IV centromere sequences.

### TgChromo1 may participate in the spatial organisation of chromosomes in the nucleus

**Figure 6:**
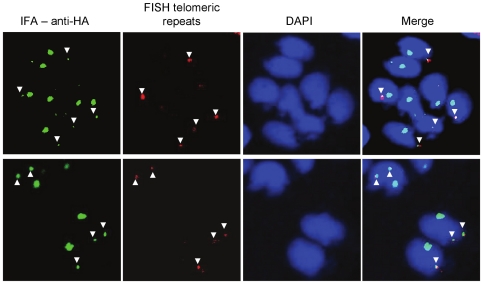
Subtelomeric repeats occupy the same nucleus territory as TgChromo1. FISH/IFA of TgChromo1-HA (green) and chromosome IX telomeric repeats (red). Parasite nuclei are labelled with DAPI (blue). Colocalising signals from FISH and IFA are arrowed.

We identified additional foci of TgChromo1 in the nucleus of *T. gondii*. Since these additional spots of concentrated TgChromo1 do not colocalise with the peri-centromeric sequences using FISH, we hypothesised that TgChromo1 might bind other sequences that were not present on the tiling microarray. Such sequences may be (i) un-assembled sequences not included in the latest version of the genome or (ii) repeated sequences that were excluded from the microarray for specificity purposes. We tested the colocalisation of the telomeric repeats with the protein, TgChromo1, using FISH and IFA and demonstrated that additional spots of TgChromo1 are in close proximity to the sub-telomeric repeats of Chromosome IX and IA (Figure 6). FISH revealed for the fist time that telomeres are clustered in the nuclear periphery of *Toxoplasma* much like *P. falciparum* [18], although *T. gondii* telomeres do not harbour antigenic variants like *P. falciparum*. However, the biological significance of this clustering remains to be explored in *T. gondii*. TgChromo1, with its specific sub-nuclear localisation, will allow a dissection of the biological relevance of chromosome clustering at the nuclear periphery of *T. gondii*.

Interestingly, HP1-like chromodomain proteins in other eukaryotes are also localised at the telomeric and subtelomeric sequences [19], including PfHP1 in *P. falciparum* [16]. The binding of HP1 to the telomeres is independent of the chromodomain and H3K9me3. This is in contrast to the binding of HP1 to the subtelomeric heterochromatin, which is dependent on the presence of H3K9me3 [19]. Surprisingly, neither TgChromo1 nor H3K9me3 are enriched at the subtelomeric regions in *T. gondii*, as measured by ChIP-on-chip, suggesting the absence of subtelomeric heterochromatin in this parasite. However, telomeric sequences might not be correctly assembled in the genome due to their repetitive nature and therefore excluded from the microarray design. TgChromo1 may bind to the telomeric sequence through a pathway independent from H3K9me3, as shown for *Drosophila* [19] and *Arabidopsis* [20] and involve indirect interactions with DNA through other proteins, thereby precluding the identification of TgChromo1 to the telomeric sequences by standard techniques.

**Figure 7:**
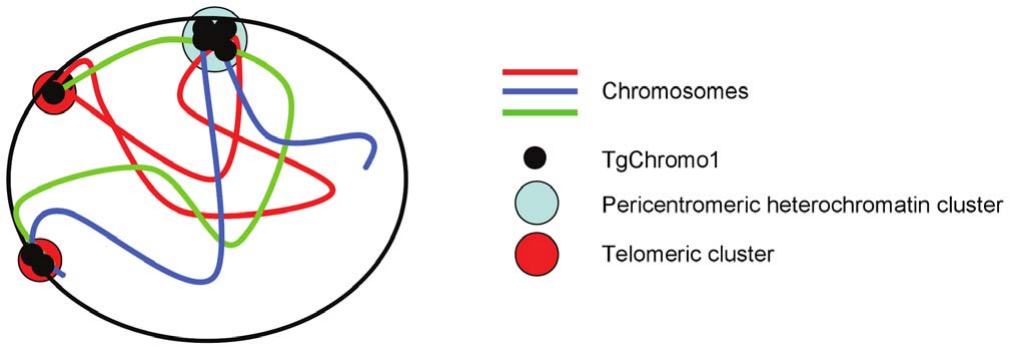
TgChromo1 participates to the nuclear organisation of the nucleus. TgChromo1 participates in the functional organisation of the nucleus. The schematic represents the chromosomes in the nucleus with the centromere and telomere clusters occupied by TgChromo1 and their position at the periphery of the nucleus.

In conclusion, we showed that the chromodomain containing TgChromo1 protein is a resident of *T. gondii* heterochromatin. TgChromo1 localises at the peri-centromeric sequences and is in close proximity to markers of the centrosome and centrocone throughout the entire cell cycle of *T. gondii*. Peri-centromeric chromatin may be involved in the migration of chromosomes toward the two mitotic poles and may participate in mitosis through TgChromo1. This is in contrast to the *Plasmodium* centromeres, which are not enriched in H3K9me3 and PfHP1 [16], underlining the specificities of *Plasmodium* epigenome. We showed that TgChromo1 may participate in the spatial organisation of the nucleus through its interaction with peri-centromeric sequences. TgChromo1 also colocalises with peri-telomeric sequences. Figure 7 summaries our findings that combined FISH and IFA to show that TgChromo1 occupies nuclear territories where peri-centromeric and telomeric regions are clustered. In *P. falciparum*, telomeres and peri-telomeric sequences harbour genes responsible for antigenic variation and cluster at the periphery of the nucleus [18]. Movement of the telomere ends in and out of those telomeric clusters may have a role in the expression of those genes [18].

In this study, we demonstrate for the first time that telomeric sequences are also sequestered to the nuclear periphery in *T. gondii*. Moreover, TgChromo1 occupies the same nuclear territory as these telomeric sequences indicating that it may participate in their sequestration to this location. The functional reason for this association is not yet understood but TgChromo1 may have a role in tethering those sequences to the nuclear membrane. Since telomeric sequences are not enriched in H3K9me3, it is unclear how TgChromo1 would bind to these regions. Chromodomain proteins have been shown in other eukaryotes to bind indirectly to telomeric sequences through their interaction with other proteins [19]. We also show that the *T. gondii* nucleus is functionally compartmentalised, with a concentration of heterochromatin at specific positions of the nuclear periphery. In eukaryotes, sub-nuclear compartments, which are not delimited by membranes, arise from the clustering of DNA sequences associated with specific proteins, thereby creating micro-environments that can favour or impede particular enzymatic activities [21]. Furthermore, we demonstrate that *T. gondii* has the ability to define chromosome territories within its nucleus, and TgChromo1 does not participate in the silencing of developmentally regulated genes (e.g. *eno1*). In the same line, H3K9me3 was not considered to be enriched at these loci [12], suggesting that heterochromatin does not have a clear role in repressing the expression of these genes. It is not yet clear how *T. gondii* represses the expression of genes. The identification of a repertoire of miRNA sequences [22] as well as the discovery of a wealth of plant-like transcription factors encompassing an AP2 domain in the *T. gondii* genome [23] may provide an explanation for the absence of heterochromatin marks at the promoters of developmentally regulated genes.

## Materials and Methods

### Ethics statement

All animal experiments were performed following the guidelines of the Institut Pasteur de Lille study board, which conforms to the Amsterdam Protocol on animal protection and welfare, a Directive 86/609/EEC for the Protection of Animals Used for Experimental and Other Scientific Purposes, updated in the Council of Europe's Appendix A (http://conventions.coe.int/Treaty/EN/Treaties/PDF/123-Arev.pdf). The animal work also complied with the French law (n°87–848 dated 19-10-1987) and the European Communities Amendement of Cruelty to Animals Act1976. All animals were fed with regular diet and all procedures were in accordance with national regulations on animal experimentation and welfare authorized by the French Ministry of Agriculture and Vetenary committee (Permit number: 59-009145). The Pasteur Institute of Lille and the CNRS Committee on the Ethics of Animal Experiments specifically approved this study.

### Parasite tissue culture and manipulation

*T. gondii* strain RH ΔKu80 [15] tachyzoites were propagated in human foreskin fibroblasts (HFF, from Millipore) using Dulbecco's modified Eagle's medium supplemented with 10% FCS (Fetal Calf Sera), 2 mM glutamine and 1% penicillin-streptomycin. *T. gondii* tachyzoites were grown in ventilated tissue culture flasks at 37°C and 5% CO_2_. Transgenes were introduced by electroporation into tachyzoites of the *T. gondii* RH ΔKu80 strain (a gift from Vern Carruthers, University of Michigan, USA) and stable transformants were selected by culture in the presence of the appropriate drugs (2 µM pyrimethamine or 25 µg/ml Mycophenolic acid and 50 µg/ml Xanthine). Clonal lines were obtained by limiting dilution.

### DNA manipulation

Target genes were amplified from genomic DNA of the parent strain and cloned into the corresponding pLIC vectors [15] kindly provided by Drs. Vern Carruthers (University of Michigan, USA) and Michael White (University of South Florida, USA). TgChromo1 (amino acid from 554 to 1084 of TGME49_068280) was cloned into the pGex6P3 plasmid in order to produce a recombinant version of the protein using *BamH1* and *EcoR1* sites. The A list of primers used is provided in Table S1.

### Recombinant protein expression and purification

*E. coli* BL21 bacteria were transformed with the pGex6P3-TgChromo1 plasmid. Bacteria were grown until they reached 0.4 OD at 600 nm and protein expression was induced using 0.1 mM IPTG overnight at room temperature. Bacteria were then lysed in a PBS containing 1% Triton X-100, 5 mg/ml lysosyme and PMSF and subjected to sonication. Soluble proteins were separated by centrifugation and added to glutathione agarose beads over-night at 4°C. The beads were washed five times with PBS. GST-tagged TgChromo1 was eluted from the column in elution buffer (50 mM Tris.HCl pH8.0 and 10 mM reduce glutathione).

### Cellular fractionation and Western blot

Intracellular parasites (5×10^8^ tachyzoites) of the TgChromo1-HA ΔKu80 RH strain were purified on a 3-µm filter and washed twice with PBS. The parasite pellet was resuspended in the 2 ml NEB1 buffer (10 mM HEPES pH 7.9, 1.5 mM MgCl_2_, 10 mM KCl, 0.5 mM DTT, 0.1 mM EDTA, 0.65% NP40 and 0.5 mM PMSF), incubated on ice for 10 min and centrifuged at 1500 g for 10 min at 4°C. The supernatant was kept as the cytoplasmic extract. The pellet was then resuspended with 400 µl of buffer NEB2 (20 mM HEPES pH 7.9, 1.5 mM MgCl_2_, 420 mM NaCl, 0.2 mM EDTA, 0.5 mM DTT, 25% glycerol and 0.2 mM PMSF) for 10 min on ice and centrifuged at 12000×g for 10 min at 4°C. The supernatant was kept as the nuclear extract. The insoluble material was extracted using an SDS buffer (2%SDS, 10 mM Tris and 0.2 mM PMSF) for 20 min at room temperature and centrifuged at 12000×g for 10 min. Protein concentration was estimated using the standard Bradford assay. Five µg of each fraction was loaded on an acrylamide gel and subjected to Western blot using the anti-HA, the anti-TgLDH1 and the anti-Histone H4 antibodies.

### Polyclonal antibodies

The anti-TgChromo1 was produced in mouse after 4 injections of 50 µg of the recombinant TgChromo1 protein. It was used at 1∶200 dilutions in IFA. The anti-MORN1 and anti-Cen1 rabbit antibodies were kindly provided by Marc-Jan Gubbels (Boston College, MA, USA) and used at 1∶1000 dilutions in IFA. The anti-IMC1 was provided by Gary Ward (University of Vermont, USA) and used at a 1∶2000 dilution in IFA. Anti-HA rabbit (Eurogentec) or mice (Invitrogen) were use at 1∶500 in IFA and 1∶2000 in Western blots.

### TgChromo1 pull-down and peptide competition

Histone peptide–binding assays were performed as described previously [14]. Briefly, 1.0 µg of a biotinylated histone peptide (Millipore) was incubated with TgChromo1 protein in binding buffer [50 mM Tris-HCl (pH 7.5), 300 mM NaCl, 0.1% Nonidet P-40, 1 mM PMSF, plus protease inhibitors] overnight at 4°C with rotation. After one hour of incubation with Streptavidin beads (Pierce) and extensive washing, bound proteins were eluted using 10-fold excess of non-biotinylated histone peptides H3K9me3, H3K9ac, H3K27me3, H3 (Diagenode) and analyzed by SDS/PAGE.

### Chromatin immunoprecipitation and microarray

Chromatin immunoprecipitation was performed as described previously [9] using either an anti-HA polyclonal rabbit antibody (Eurogentec) or a polyclonal mouse antibody that was raised against the recombinant version of TgChromo1.

A tilling microarray was designed by Genotypic Technology (India) based on the version 6 of the *T. gondii* ME49 genome (available at www.toxodb.org) and printed by Agilent Technologies. The microarray encompasses more than 983000 features representing all the genome with an average coverage of one oligonucleotide every 63 bp. Purified ChIP material was processed according to the Agilent Mammalian ChIP-on-chip protocol version 10.11, and labelled DNA was hybridized to Agilent *T. gondii* tiling array for 40 hours at 65°C (G4481-90010; Agilent Technologies,). Microarrays were washed and scanned as per manufacturer's protocol and results were processed with the Genomic workbench Standard edition.

### Fluorescent in situ hybridization

Intracellular parasites were fixed with 4% paraformaldehyde after 24 hours infection and processed according to previously published protocols [24]. Probes were labelled according to Muller *et al.* [25]. Probes were denatured at 95°C for 5 min and then incubated on ice for 2 min. Denaturation of parasite DNA (80°C for 15 min) and hybridization (overnight at 37°C) was made on an Eppendorf thermocycler using a slide adaptator. Coverslips were washed 15 min at 50°C with 1× SSC followed with 2 washes with 2× SSC and 4× SSC in the same condition and mounted with Mowiol on slides.

For FISH-IFA, the same protocol was followed by incubation in IFA blocking buffer (0.2% Triton X-100, 3% FBS). Primary antibody was incubated for 1 hour at room temperature. Secondary antibodies coupled to Alexa Fluor-488 was incubated 1 hour together with DAPI at room temperature and mounted with Mowiol on slides.

### Imaging by confocal microscopy

Confocal imaging was performed with a LSM710 microscope (Zeiss) and a Plan Apochromat objective (Plan-Apochromat 63×/1.40 Oil DIC M27, Zeiss) as previously described [26]. The associated software (Zen 2008) enabled the adjustment of acquisition parameters. Fluorescent signals were collected sequentially, with a 4 lines average, a zoom factor (varying between 2 and 4) and resulting images are 512×512 pixels in size, and 8 bits in resolution (256 gray levels). By setting the photomultiplier tubes and the pinhole size (1 AU) correctly, there was no signal bleed-through. The images were treated with ImageJ (NIH). Z-stack acquisitions enabled to visualize the 3D localization of fluorescent signals. Rotation (360° around the y axis) movies were created with the Zen software, at a rate of 5 frames per second. The percentage of overlapping signals was measured by the co-localisation tool of the Zen software on more than 100 parasites.

## Supporting Information

Figure S1 **Multiple sequence alignment of TgChromo1 chromodomain with other chromodomain containing proteins.** Alignment was performed with Multialign (http://multalin.toulouse.inra.fr/multalin/) aligning chromodomain amino-acid sequences of *S. pombe* Swi6 (Sp); *D. discoideum* HP1α (Dd); *N. crassa* HP1 (Nc); *H. sapiens* HP1α (Hs); Mm, *D. melanogaster* Su (var)205 (Dm); *C. elegans* HP1-like (Ce); *P. falciparum* PfHP1 (Pf) and TgChromo1. Residues important for contacting and binding to H3K9me3 are highlighted in yellow. Conserved residues are in red. Residues are considered low consensus (in blue) when they appear in less than 90% and more than 50% of the sequences. Consensus symbols: ! is anyone of the amino-acids I or V, $ is anyone of the aa L or M, % is anyone of the aa F or Y, # is anyone of the aa N or D or Q or E.
(PPT)

Figure S2 **Allelic replacement at the TgChromo1 endogenous loci.** **A:** Schematic representing the native and recombinant TgChromo1 locus. Primer couples used as positive control (AB) and for screening of the recombinant (CD) are presented. **B:** Ethidium-bromide dyed gel after PCR of the primers AB and CD for WT and recombinant strains.
(PPT)

Figure S3 **TgChromo1 is not expressed in the majority of extracellular parasites.** Extracelllular parasites were fixed and subjected to IFA using an anti-HA (green) antibody. Parasite nuclei are labelled with DAPI (blue).
(PPT)

Figure S4 **An antibody against the TgChromo1 recombinant protein confirm the cell cycle regulation of its expresssion.** Intracellular parasites were fixed and subjected to IFA using an anti-TgChromo1 (green) antibody and anti-TgMORN1 antibody (red), a marker of the centrocone. Parasite nuclei are labelled with DAPI (blue). Parasites during mitosis express TgChromo1 when this protein is undetectable toward the end of the budding or the beginning of G1.
(PPT)

Figure S5 **Specificity of the TgChromo1 antibody.** Cytoplasmic, nuclear and insoluble extracts from the RH ΔKu80 strain were subjected to a Western-blot using the anti-TgChromo1 specific antibody. A single band is identified in the nuclear and insoluble material.
(PPT)

Figure S6 **Real-time quantitative PCR validation of ChIP on chip results.** Relative enrichment is represented as a ratio of the signal given by the immunoprecipitated DNA compared to the input DNA. Eight loci were tested for enrichment: the silenced *eno1* promoter (eno1), the active *eno2* promoter (eno2), centromeric chromatin at chromosome Ia (CIa), pericentromeric heterochromatin at chromosome Ia (PcIa), centromeric chromatin at chromosome VIII (CVIII), pericentromeric heterochromatin at chromosome Ia (PcVIII), centromeric chromatin at chromosome Ia (CXI) and pericentromeric heterochromatin at chromosome Ia (PcXI). Error bars represent standard deviation for two independent experiments.
(PPT)

Movie S1 **3D reconstruction using sequential z-stack series demonstrates the localisation of TgChromo1 to perinuclear clusters.**
(AVI)

Table S1 **Primer used in this study.**
(XLS)
