# Supplementary figures and images for: Toxoplasma gondii Chromodomain Protein 1 Binds to Heterochromatin and Colocalises with Centromeres and Telomeres at the Nuclear Periphery
Source: PLoS One. 2012 Mar 9;7(3):e32671. doi: 10.1371/journal.pone.0032671 (PMC3302879; doi:10.1371/journal.pone.0032671)

## Slide 1
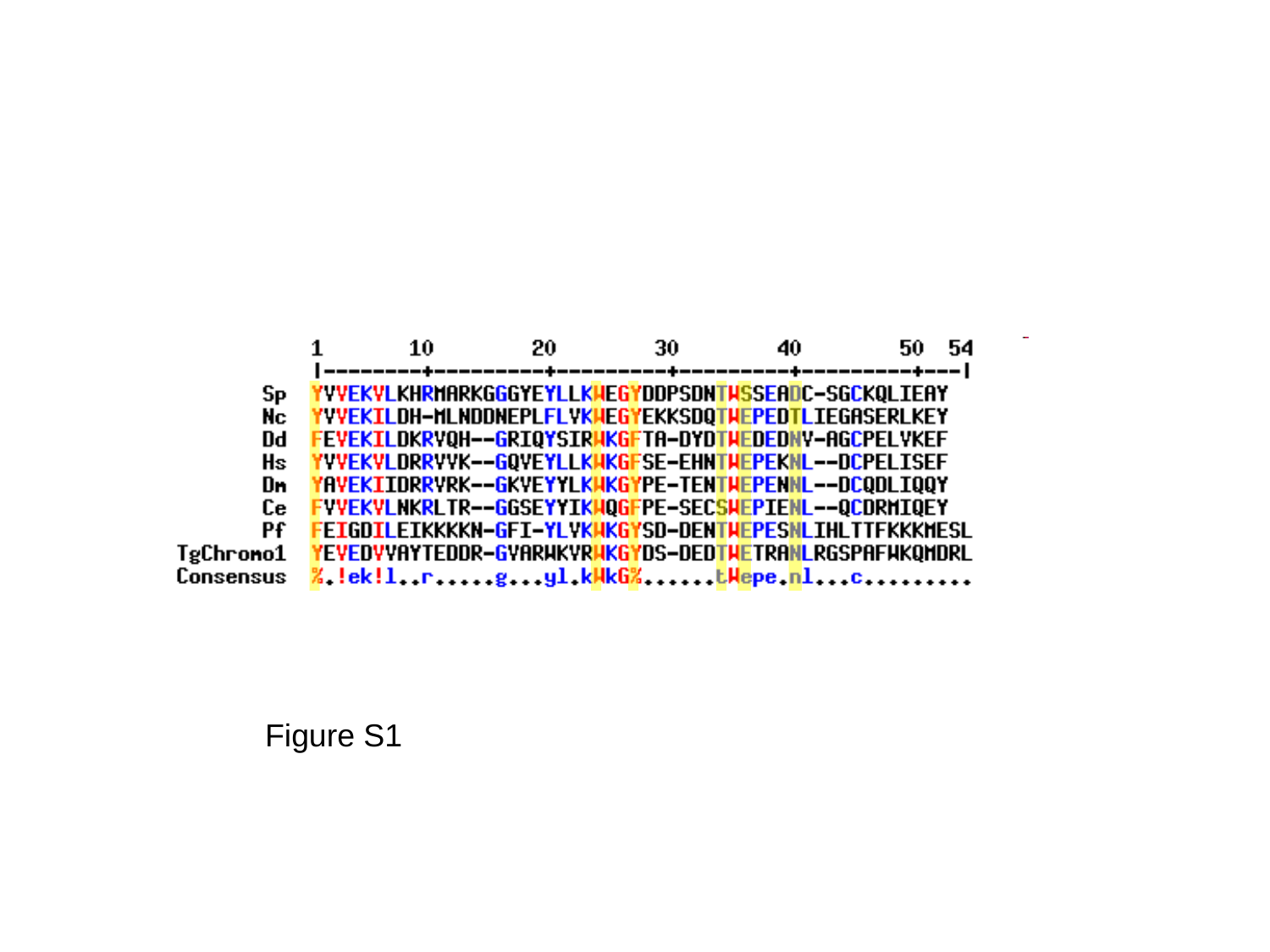

Figure S1

Supplement: Figure S1 — Multiple sequence alignment of TgChromo1 chromodomain with other chromodomain containing proteins. Alignment was performed with Multialign (http://multalin.toulouse.inra.fr/multalin/) aligning chromodomain amino-acid sequences of S. pombe Swi6 (Sp); D. discoideum HP1α (Dd); N. crassa HP1 (Nc); H. sapiens HP1α (Hs); Mm, D. melanogaster Su (var)205 (Dm); C. elegans HP1-like (Ce); P. falciparum PfHP1 (Pf) and TgChromo1. Residues important for contacting and binding to H3K9me3 are highlighted in yellow. Conserved residues are in red. Residues are considered low consensus (in blue) when they appear in less than 90% and more than 50% of the sequences. Consensus symbols: ! is anyone of the amino-acids I or V, $ is anyone of the aa L or M, % is anyone of the aa F or Y, # is anyone of the aa N or D or Q or E. (PPT) [file pone.0032671.s001.ppt]

## Slide 1
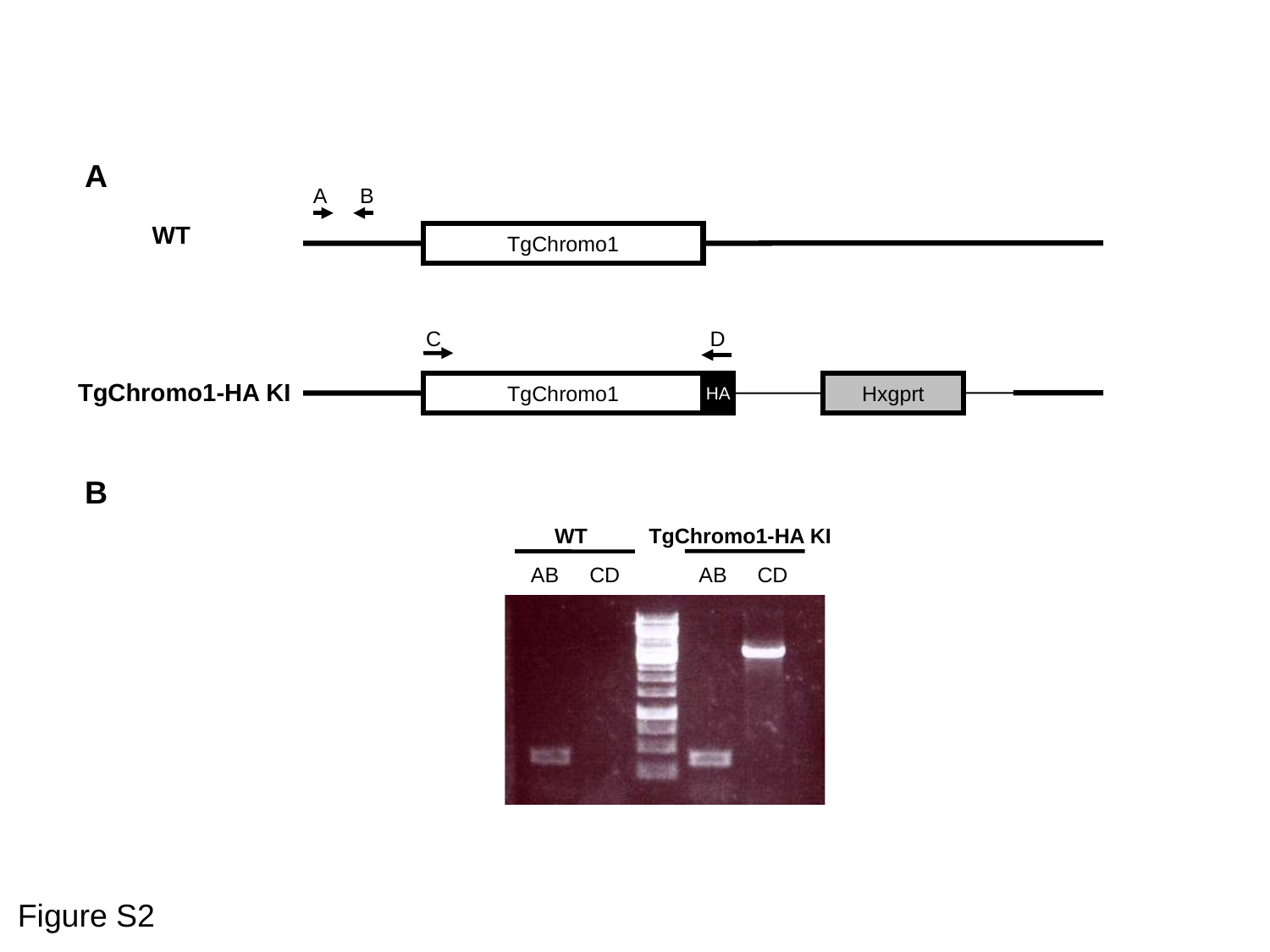

A
A
B
TgChromo1
WT
C
D
TgChromo1
HA
Hxgprt
TgChromo1-HA KI
B
TgChromo1-HA KI
WT
AB
CD
AB
CD
Figure S2

Supplement: Figure S2 — Allelic replacement at the TgChromo1 endogenous loci. A: Schematic representing the native and recombinant TgChromo1 locus. Primer couples used as positive control (AB) and for screening of the recombinant (CD) are presented. B: Ethidium-bromide dyed gel after PCR of the primers AB and CD for WT and recombinant strains. (PPT) [file pone.0032671.s002.ppt]

## Slide 1
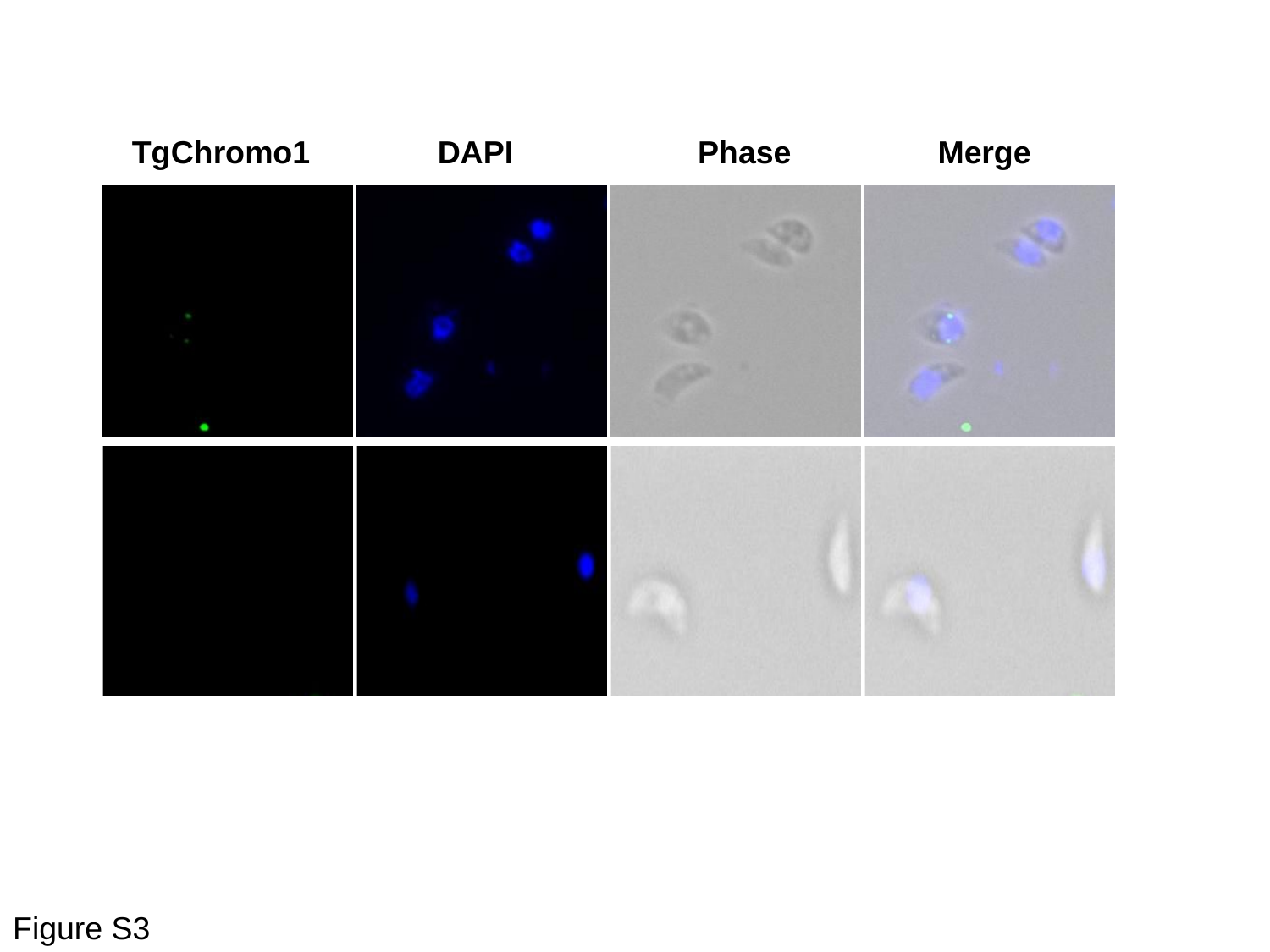

TgChromo1
DAPI
Phase
Merge
Figure S3

Supplement: Figure S3 — TgChromo1 is not expressed in the majority of extracellular parasites. Extracelllular parasites were fixed and subjected to IFA using an anti-HA (green) antibody. Parasite nuclei are labelled with DAPI (blue). (PPT) [file pone.0032671.s003.ppt]

## Slide 1
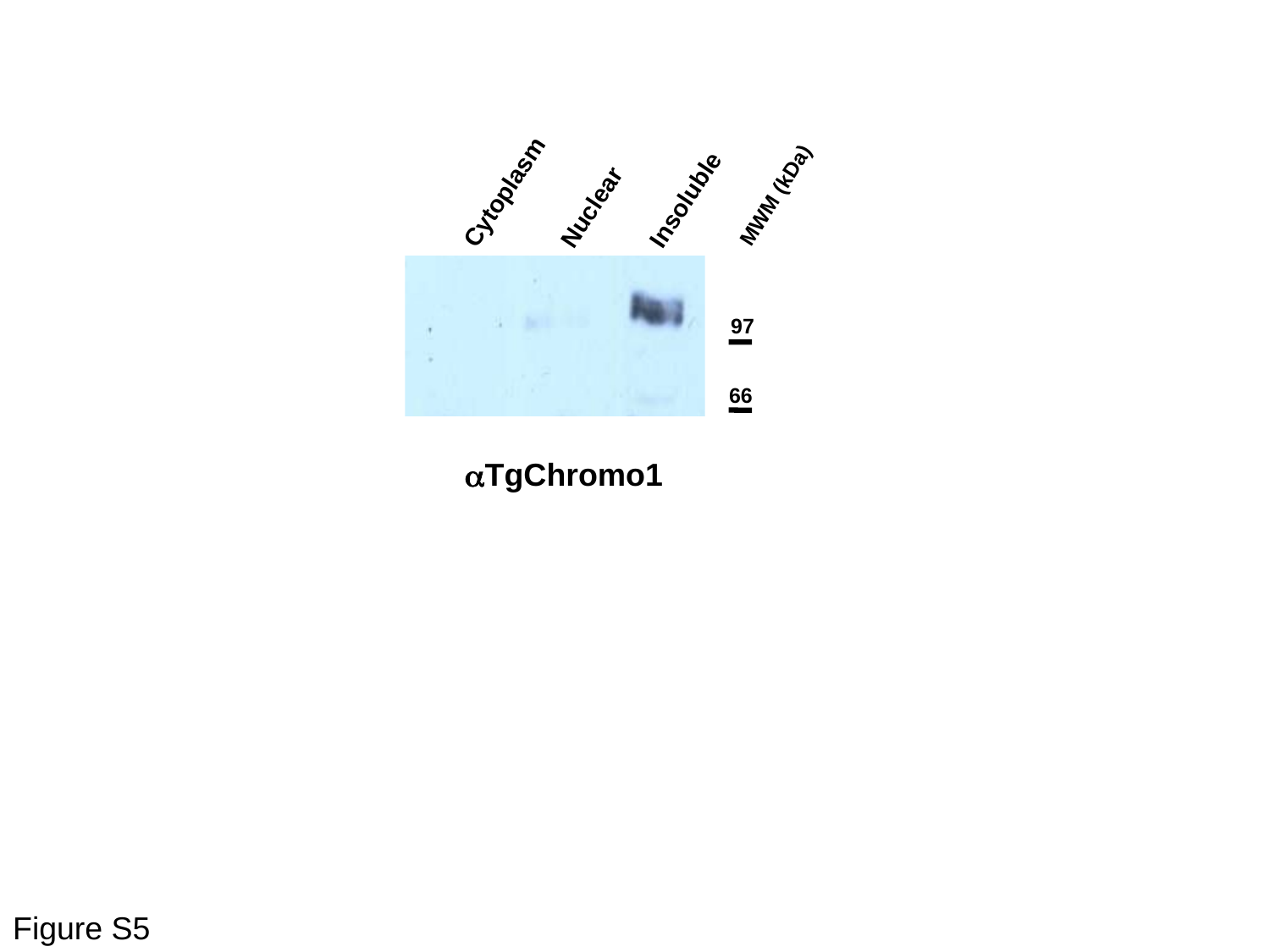

Cytoplasm
MWM (kDa)
Insoluble
Nuclear
97
66
αTgChromo1
Figure S5

Supplement: Figure S5 — Specificity of the TgChromo1 antibody. Cytoplasmic, nuclear and insoluble extracts from the RH ΔKu80 strain were subjected to a Western-blot using the anti-TgChromo1 specific antibody. A single band is identified in the nuclear and insoluble material. (PPT) [file pone.0032671.s005.ppt]

## Slide 1
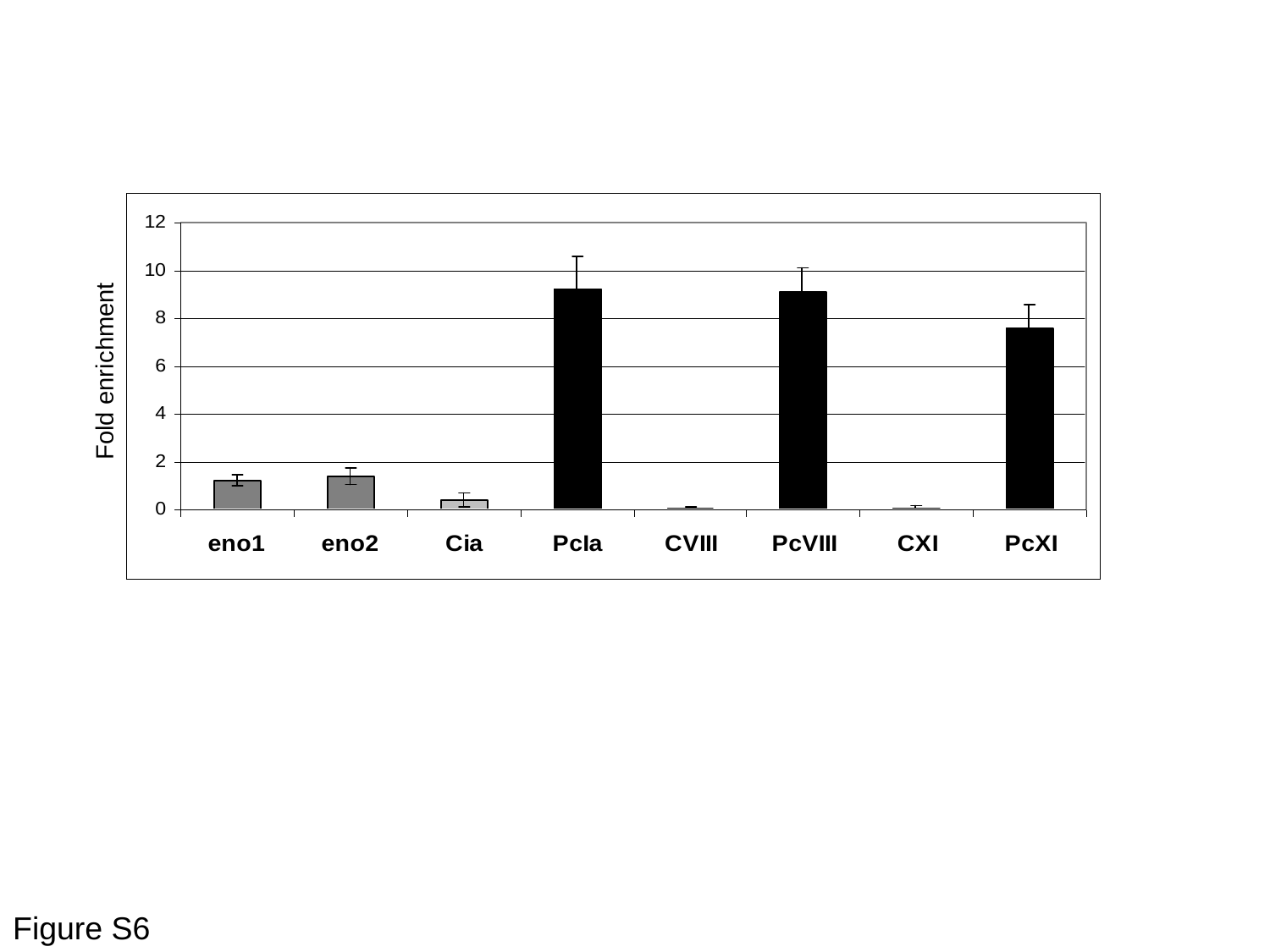

Fold enrichment
Figure S6

Supplement: Figure S6 — Real-time quantitative PCR validation of ChIP on chip results. Relative enrichment is represented as a ratio of the signal given by the immunoprecipitated DNA compared to the input DNA. Eight loci were tested for enrichment: the silenced eno1 promoter (eno1), the active eno2 promoter (eno2), centromeric chromatin at chromosome Ia (CIa), pericentromeric heterochromatin at chromosome Ia (PcIa), centromeric chromatin at chromosome VIII (CVIII), pericentromeric heterochromatin at chromosome Ia (PcVIII), centromeric chromatin at chromosome Ia (CXI) and pericentromeric heterochromatin at chromosome Ia (PcXI). Error bars represent standard deviation for two independent experiments. (PPT) [file pone.0032671.s006.ppt]
