# Supplementary material for: Toxoplasma gondii Chromodomain Protein 1 Binds to Heterochromatin and Colocalises with Centromeres and Telomeres at the Nuclear Periphery
Source: PLoS One. 2012 Mar 9;7(3):e32671. doi: 10.1371/journal.pone.0032671 (PMC3302879; doi:10.1371/journal.pone.0032671)
Supplement: Figure S4 — An antibody against the TgChromo1 recombinant protein confirm the cell cycle regulation of its expresssion. Intracellular parasites were fixed and subjected to IFA using an anti-TgChromo1 (green) antibody and anti-TgMORN1 antibody (red), a marker of the centrocone. Parasite nuclei are labelled with DAPI (blue). Parasites during mitosis express TgChromo1 when this protein is undetectable toward the end of the budding or the beginning of G1. (PPT) [file pone.0032671.s004.ppt]

## Slide 1
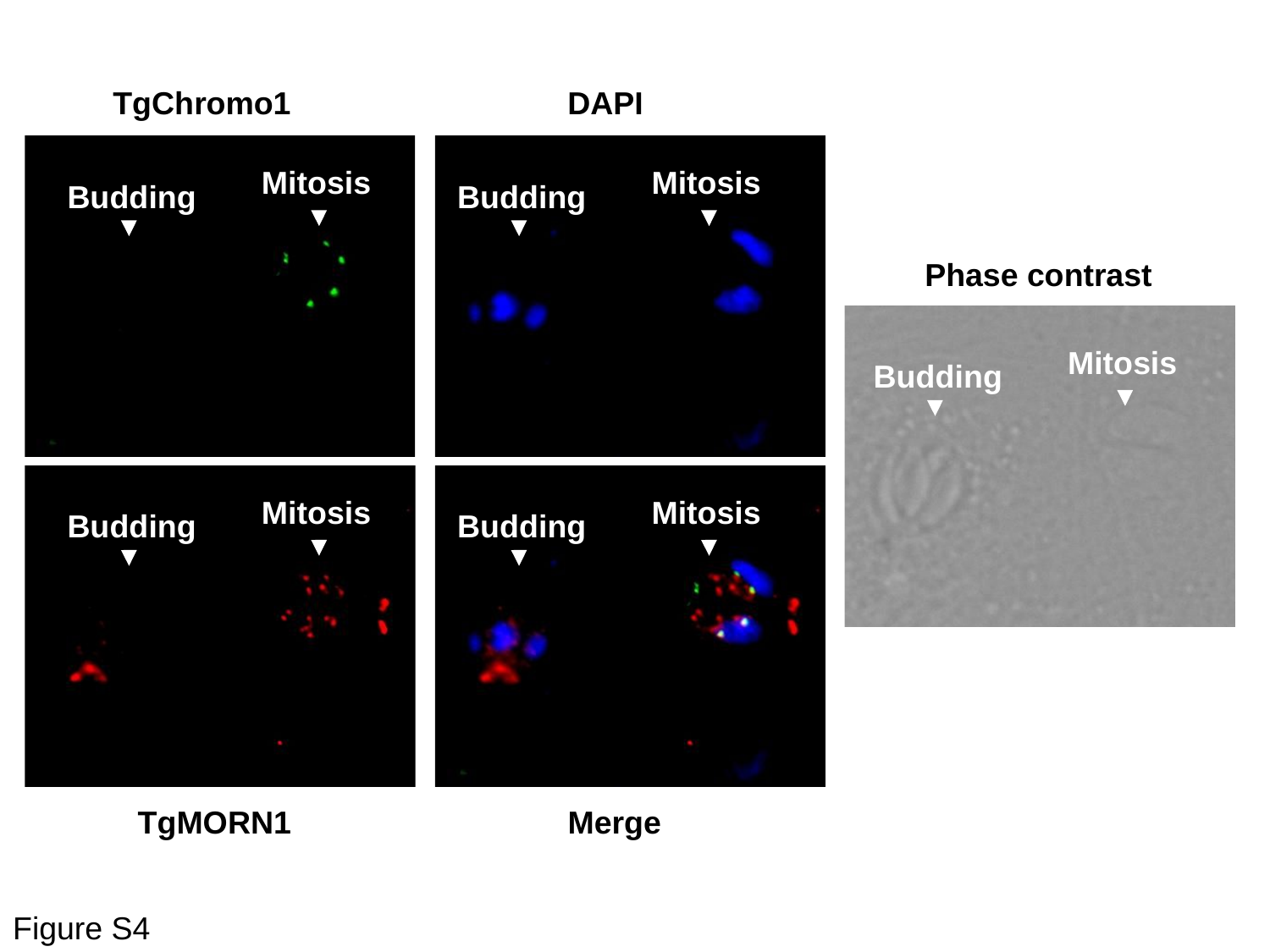

TgChromo1
DAPI
C
S
C
S
Mitosis
Budding
Mitosis
Budding
Phase contrast
Mitosis
Budding
Mitosis
Budding
Mitosis
Budding
C
S
TgMORN1
Merge
Figure S4
